# Lard intake results in better hypothalamic leptin responsiveness than beef tallow intake during overnutrition

**DOI:** 10.1371/journal.pone.0326847

**Published:** 2025-07-21

**Authors:** Mutsumi Ikeda, Tamae Shiino, Kai Naruke, Nozomi Takahashi, Hanako Kanzaki, Ran Xu, Toshimi Shirozu, Misato Nakano, Miharu Murae, Yui Funatsu, Kentaro Kaneko

**Affiliations:** Department of Agricultural Chemistry, School of Agriculture, Meiji University, 1-1-1, Higashimita, Tama-ku, Kawasaki-shi, Kanagawa 214, Japan; Auburn University, UNITED STATES OF AMERICA

## Abstract

The hypothalamus helps regulate energy homeostasis, but high-fat diet intake leads to body weight gain and diminishes hypothalamic responses to metabolic hormones such as leptin. The fatty acid compositions of beef tallow and lard, which are representative animal fats in experimental diets, are similar. However, it is not clear how differences in the effects of obesogenic conditions induced by the intake of beef tallow or lard affect hypothalamic leptin responsiveness. Herein, we showed that under obesogenic conditions as 45 *kcal% fat*, lard-fed mice had lower body weight gain, food intake, respiratory quotient, and adiposity and better glucose balance and insulin sensitivity than beef tallow-fed mice. Furthermore, the central administration of leptin reduced body weight and food intake in mice fed lard, but not beef tallow, under the same overnutrition conditions. Lard-fed mice also exhibited lower levels of hypothalamic suppressor of cytokine signaling-3, an inhibitor of leptin activity, than mice fed beef tallow. While there were no differences in body weight with the low-fat diet as 30 *kcal% fat*, central leptin-induced body weight and food intake reductions in lard were higher than those in beef tallow. Finally, we showed that leptin-deficient *ob/ob* mice, another mouse model of obesity, did not show any improvement in energy balance under lard-fed conditions. Our results showed that lard was better than beef tallow in maintaining hypothalamic leptin responsiveness and systemic metabolic states under obesogenic conditions.

## Introduction

Obesity is globally recognized as a major health concern in children and adults and is associated with serious comorbidities, including type 2 diabetes, hypertension, cardiovascular disease, certain cancers, and reduced life expectancy. A reduction in body weight or protection against body weight gain has a beneficial impact on several metabolic risk factors.

The hypothalamus, located within the central nervous system, is an important region that controls energy balance and maintains normal body weight and feeding behavior by sensing nutrients and appetite-regulating hormones. Leptin is a powerful adipocyte-derived satiety hormone that acts on the hypothalamus through the leptin receptor, which helps to normalize body weight by regulating food intake and energy expenditure [1–6].

Excess nutrients, which can result from consuming a high-fat diet, rapidly reduce hypothalamic responses to exogenously administered leptin and induce dietary obesity [4–6]. High-fat diet intake blunted leptin-dependent hypothalamic phosphorylation of signal transducer and activator of transcription 3 (pSTAT3), a critical mediator of leptin actions [7–10]. A major signaling molecule that directly inhibits leptin signaling by hypothalamic induction is suppressor of cytokine signaling-3 (SOCS3) [11–13]. Because decreased leptin responsiveness is a major cause of obesity under high-fat diet-induced overnutrition conditions [1–6], enhanced or maintained hypothalamic leptin responsiveness can protect against dietary obesity and metabolic disruption by controlling food intake and maintaining leptin and insulin sensitivity [14–19].

In laboratory animal research, beef tallow and lard are used as representative animal fats in high-fat diet formulas. Palmitic acids are the most abundant saturated and unsaturated fatty acids in beef tallow and lard [20]. The fatty acid compositions of beef tallow and lard are similar [20]. Both beef tallow and lard contain approximately 24% palmitic acid; however, more than 70.5 mol% of palmitic acid in lard is located at the sn-2 position, whereas only ~15 mol% of palmitic acid in beef tallow is located at the sn-2 position [21–23]. We have recently shown that 2-monopalmitin, also known as β-palmitate, but not 1-monopalmitin, plays an important role in the protection against high-fat diet (HFD)-induced decreased leptin responsiveness [24]. When palmitic acid binds to the sn-2 or beta position of the glycerol backbone, it is known as 2-monopalmitin or β-palmitate. This study also demonstrated the anti-obesity and anti-diabetic effects of 2-monopalmitin under overnutritional conditions [24]. Thus, we propose that 2-monopalmitin may be a valuable therapeutic agent to alleviate obesogenic conditions. Because lard has a higher 2-monopalmitin content than beef tallow, we hypothesized that lard intake might better maintain hypothalamic leptin responsiveness than beef tallow intake under overnutrition conditions. However, it is unclear whether beef tallow or lard is better at maintaining hypothalamic leptin responsiveness under these conditions. In this study, we investigated the effects of overnutrition caused by lard and beef tallow intake on hypothalamic leptin responsiveness and energy balance.

## Materials and methods

### Animals and diets

Male C57BL/6 (3 weeks old) and *ob/ob* mice were purchased from Japan SLC (Shizuoka, Japan). Mice were housed under constant temperature (22–24°C) and humidity (50–60%) using 12-h light and dark cycles (lights on 7 a.m.–7 p.m.). Mice were randomly divided into lard-fed and beef tallow-fed groups after one week of adaptation, with *ad libitum* access to water and a lard or beef tallow diet (45 *kcal%* or 30 *kcal%* *fat*). Lard and beef tallow diets (Oriental Yeast Co. LTD, Tokyo, Japan) were prepared as described in Table 1. The mice were weighed once weekly. At the end of the experiment, the mice were euthanized by cervical dislocation after isoflurane inhalation. All experimental procedures were conducted according to the ARRIVE guidelines. The care of all animals and the procedures were approved by the Animal Committee of the Meiji University (MUIACUC2022−05).

**Table 1:** Composition of main ingredients and principal component comparison in lard and beef tallow diets.

Raw materials name		Unit	EE Kcal 45% Lard	EE Kcal 30% Lard	EE Kcal 45% Beef tallow	EE Kcal 30% Beef tallow
Casein		%	14.00	14.00	14.00	14.00
Cystine		%	0.18	0.18	0.18	0.18
βCorn starch		%	26.0655	37.3674	26.0655	37.3674
αCorn starch		%	17.50	15.50	17.50	15.50
Sucrose		%	10.00	10.00	10.00	10.00
Soybean oil		%	4.00	2.00	4.00	2.00
Lard		%	18.50	11.20	–	–
Beef tarrow		%	–	–	18.50	1120
Cellulose powder		%	4.00	4.00	4.00	4.00
AIN-93M mineral mixture		%	3.50	3.50	3.50	3.50
AIN-93 vitamin mixture		%	1.00	1.00	1.00	1.00
Choline bis(tartrate)		%	0.25	0.25	0.25	0.25
Tertiary butylhydroquinone		%	0.0045	0.0026	0.0045	0.0026
Total		%	100.00	100.00	100.00	100.00
Nutritional ingredients						
Moisture content		%diet	9.0	9.0	9.0	9.0
Crude protein		%diet	12.4	12.6	12.4	12.6
Crude fat		%diet	22.1	13.3	22.1	13.3
Crude ash		%diet	2.9	3.0	2.9	3.0
Crude fiber		%diet	4.9	5.0	4.9	5.0
NFE		%diet	48.7	57.2	48.7	57.2
Total calories		kcal/100 g diet	443.1	398.7	443.1	398.7
Protein calorie ratio		%kcal	11.2	12.6	11.2	12.6
Lipid calorie ratio		%kcal	44.9	30.0	44.9	30.0
NFE calorie ratio		%kcal	44.0	57.4	44.0	57.4
	Lauric acid	%TFA	0.16	0.17	0.08	0.08
	Myristic acid	%TFA	1.42	1.46	2.07	2.14
	Pentadecanoic acid	%TFA	0.08	0.08	0.25	0.25
	Palmitic acid	%TFA	22.52	22.90	23.34	23.75
	Palmitoleic acid	%TFA	2.07	2.14	2.48	2.56
	Stearic acid	%TFA	12.60	12.87	13.67	13.97
	Oleic acid	%TFA	39.70	40.22	41.59	42.17
	Linoleic acid	%TFA	17.40	16.25	12.55	11.25
	α-Linolenic acid	%TFA	1.58	1.42	1.34	1.17
	Arachidic acid	%TFA	0.24	0.23	0.15	0.15
	Icosenoic acid	%TFA	0.61	0.62	0.36	0.37
	Icosapentaenoic acid	%TFA	0.00	0.00	0.00	0.00
	Behenic acid	%TFA	0.07	0.06	0.07	0.06
	Docosapentaenoic acid	%TFA	0.00	0.00	0.00	0.00
	Docosahexaenoic acid	%TFA	0.00	0.00	0.00	0.00
	Lignoseric acid	%TFA	0.02	0.02	0.02	0.02
	Tetracosenoic acid	%TFA	0.00	0.00	0.00	0.00
Other		%TFA	1.52	1.56	2.01	2.07
Saturated fatty acids		%TFA	37.11	37.79	39.66	40.42
Monounsaturated fatty acids		%TFA	42.38	42.98	44.44	45.10
Polyunsaturated fatty acids		%TFA	18.99	17.68	13.89	12.42

### Physiological measurements

Body weight was measured at the indicated time points. For energy expenditure measurements, mice were acclimatized to metabolic cages and housed individually for 2–3 days before measurements were obtained. Metabolic parameters, including O_2_ consumption, CO_2_ production, respiratory exchange ratio, heat production, and ambulatory activity, were determined using the Columbus Instruments Comprehensive Lab Animal Monitoring System (CLAMS) (Columbus Instruments, Ohio, USA), as previously described [15]. Body composition was measured using a cone-beam flat-panel DXA detector (iNSiGHT VET DXA; OsteoSys, Seoul, Korea). The mice were immobilized using gas inhalation (isoflurane). Fat and lean masses were quantified based on the attenuation of two different X-ray energy levels. Food intake was measured using the MFD-100 system (Shinfactory, Fukuoka, Japan). Ambulatory activity was measured using an ACTIMO system (Shinfactory, Fukuoka, Japan). Blood glucose levels were determined in freshly withdrawn blood from the tail vein using a OneTouch Ultra Blood Glucose Meter. Glucose tolerance tests were performed in overnight-fasted mice. D-glucose (1.5 g/kg) was injected intraperitoneally, and blood glucose was measured at the indicated time points from the tail vein. Insulin tolerance tests were performed on 3-hour fasted mice. Insulin (1 U/kg) was injected intraperitoneally, and blood glucose levels were measured at the indicated time points.

### Cannula implantation and leptin sensitivity test

Cannula implantation was performed as described previously [15–19]. Mice were anesthetized with isoflurane and placed in a stereotaxic frame. A 26-gauge single stainless-steel guide cannula (C315GS-5-SPC, Plastics One, Roanoke, VA, USA) was implanted into the lateral ventricles (−0.45 mm from bregma, ± 0.9 mm lateral and −2.5 mm from the skull). The cannula was fixed to the skull using screws and dental cement. The mice were housed in single cages and allowed to recover for one week. The placement of the guide cannula was verified histologically at the end of the experiment. For the leptin sensitivity test, after four months of lard or beef tallow feeding, an intracerebroventricular (ICV) cannula was implanted, and the mice were allowed to recover for one week. On experimental days, as previously described [19], mice were infused with 1 μL of the leptin solution (0.5 μg/mouse) once a day at 5 p.m. for four consecutive days. The food intake and body weight were measured daily.

### Total protein extraction and western blot analysis

Western blot analysis was performed as previously described with slight modifications [15–19]. Briefly, proteins were extracted by homogenizing the samples in lysis buffer (25 mM Tris-HCl at pH 7.4, 150 mM NaCl, 1% NP-40, 1 mM EDTA, and 5% glycerol) (87787 and 87788 Pierce IP Lysis Buffer, Thermo Fisher Scientific) containing protease and phosphatase inhibitor cocktails (1:100, 78442, Thermo Fisher Scientific). The hypothalamus was collected as follows: after brief anesthetization with isoflurane, the mice were decapitated, and the whole brain was removed. The hypothalamus was prepared using a brain matrix (1 mm thick), frozen immediately in liquid nitrogen, and stored at –80°C. Equal amounts of samples were separated by sodium dodecyl sulfate-polyacrylamide gel electrophoresis and transferred onto a nitrocellulose membrane by electroblotting. The following primary antibodies were used for western blotting: anti-phosphorylated STAT3 antibody (1:1000; Cell Signaling Technology, 9131) and anti-STAT3 antibody (1:2000; Cell Signaling Technology, 4904). After incubation with the primary antibodies for 72 h at 4°C, the membranes were incubated with gentle agitation for 1 h at room temperature using the following secondary antibodies conjugated to a chemiluminescent entity: anti-rabbit IgG and HRP-linked antibodies (1:5000; Cell Signaling Technology, 7074). WSE-6100 Lumino-GraphⅠ (ATTO Corporation, Tokyo, Japan) was used to measure the chemiluminescence intensity.

### Enzyme-linked immunosorbent assay (ELISA) experiments

Blood samples were collected from 4-hour-fasted mice. Plasma was isolated after centrifugation at 2000 rpm for 10 min and stored at −80°C. To analyze the plasma concentrations of leptin in mouse blood samples, an ELISA was performed. The ELISA kits for leptin (M1108, Morinaga Institute of Biological Science, Inc. Japan), insulin (M1305, Morinaga Institute of Biological Science, Inc. Japan), and glucagon-like peptide-1 (GLP-1) (EIAM-GLP-1; Ray Biotech, Georgia, USA) were used according to the manufacturer’s recommendations.

### Total ribonucleic acid (RNA) extraction and quantitative real-time polymerase chain reaction (PCR)

Quantitative real-time PCR analysis was performed using a previously reported method with slight modifications [15–19]. Hypothalamic samples were collected from C57BL/6 mice fed with lard or beef tallow for five months, and total RNA was extracted using an RNeasy Mini Kit (Qiagen, Hilden, Germany). Complementary deoxyribonucleic acid was generated using the Takara Prime Script® RT Master Mix (Takara, Osaka, Japan). For quantitative real-time PCR, we amplified complementary deoxyribonucleic acid using a CFX Connect Real-Time PCR Detection System (Bio-Rad Laboratories, Inc. California, USA) with the THUNDERBIRD® qPCR Mix (Toyobo Co., Osaka, Japan), and each primer was set specific for mouse cyclophilin, SOCS3, protein tyrosine phosphatase 1B (PTP1B), and T-cell protein tyrosine phosphatase (TCPTP), according to the manufacturer’s instructions, as previously described. Reactions were cycled 40 times for denaturation at 95°C for 15 s and annealing and elongation at 60°C for 60 s. Normalized mRNA levels were expressed in arbitrary units obtained by dividing the averaged, efficiency-corrected values for sample mRNA expression by that for β-actin RNA expression for each sample. The resulting values were expressed as fold-change above the average control levels. The following primer sequences were used: SOCS3 (F-CACCTGGACTCCTATGAGAAAGTG and R-GAGCATCATACTGATCCAGGAACT), PTP1B (F-GGAACAGGTACCGAGATGTCA

and R-AGTCATTATCTTCCTGATGCAATT), TCPTP (F-AGGGCTTCCTTCTAAGG

and R-GTTTCATCTGCTGCACCTTCTGAG), Agouti-related protein (AgRP)(F-CGGCCACGAACCTCTGTAG and R-CTCATCCCCTGCCTTTGC), proopiomelanocortin (POMC) (FGAGGCCACTGAACATCTTTGTC and R-GCAGAGGCAAACAAGATTGG), neuropeptide Y (NPY) (F-TCCGCTCTGCGACACTAC and R-GGGACAGGCAGACTGGTT), or β-actin (F-CTGCGCAAGTTAGGTTTTGTCA and R-TGCTTCTAGGCGGACTGTTACTG).

### Statistical analysis

All data are expressed as mean ± SEM. Statistical analyses were performed using GraphPad Prism 10 for a two-tailed unpaired Student’s t-test or one- or two-way ANOVA followed by post hoc analysis. P < 0.05 was considered statistically significant.

### Study approval

All procedures used to maintain and evaluate the mice followed protocols reviewed and approved by the Animal Research Committee of Meiji University (MUIACUC2022−05) (Kanagawa, Japan). The animal experimentation guidelines were followed.

## Results

### Lard-fed mice showed a lower body weight gain than beef tallow-fed mice under overnutrition conditions

**Fig 1:**
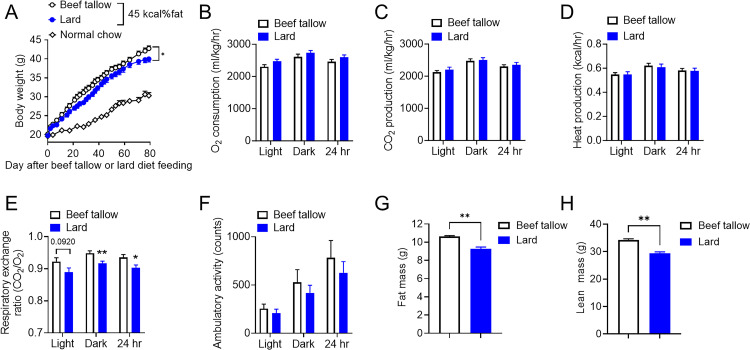
Lard intake lowered body weight gain compared with beef tallow intake under overnutrition conditions. (A–H) Metabolic phenotypes of beef tallow-fed and lard-fed fat mice. (A) Daily body weights of beef tallow- and lard-fed males over the indicated period (n = 10/group). (B–F) Metabolic profiles of beef tallow- and lard-fed male mice (n = 6/group) for O_2_ consumption (B), CO_2_ production (C), heat production (D), respiratory exchange ratio (E), and ambulatory activity (F) during the 12-h light and dark phases. Age- and body-weight-matched cohorts were considered (lard-fed: 46.98 ± 1.96 g vs. beef tallow-fed: 48.42 ± 1.44 g, p > 0.05, t-tests) in the CLAMS study. Body composition showing weight (g) of fat (G) and lean (H) mass. *p < 0.05, **p < 0.01 for two-way ANOVA followed by Sidak’s multiple comparisons tests in (A) and t-tests in (E), (G), and (H). Data represent the mean ± SEM.

Although high-fat diet feeding generally causes body weight gain and diminishes hypothalamic sensitivity to leptin, it remains unclear which types of animal fat intake adversely affect energy balance and hypothalamic leptin responsiveness. To directly evaluate this, beef tallow and lard were used as representative animal fats in the experimental diets with 45 *kcal% fat* (Table 1). To examine the effect of animal fat on energy homeostasis, mice were fed these diets starting at 4 weeks of age. Lard-fed mice showed a lower body weight gain than beef tallow-fed mice (Fig 1A). We investigated the basis for the leaner phenotype of lard-fed mice by directly assessing their energy balance using open-circuit indirect calorimetry cages. Although there were no significant differences in O_2_ consumption, CO_2_ production, heat production, or the amount of activity between lard-fed and beef tallow-fed mice, the respiratory exchange ratio was significantly lower during the dark phase in lard-fed mice than in tallow-fed mice in body weight- and age-matched cohorts (Fig 1B–F). Furthermore, lard-fed mice showed significantly lower adiposity than beef tallow-fed mice (Fig 1G and H). Overall, we confirmed that lard-fed mice showed significantly lower body weight gain and respiratory exchange ratios than beef tallow-fed mice.

### Lard-fed mice showed better glucose balance and insulin sensitivity than beef tallow-fed mice under overnutrition conditions

**Fig 2:**
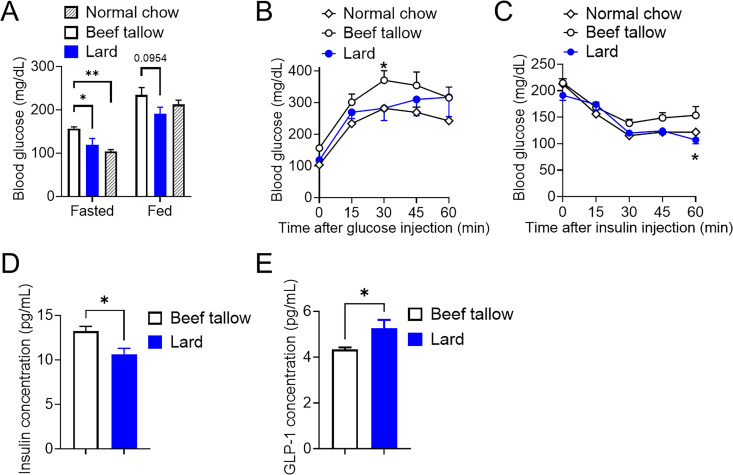
Lard intake shows better glucose homeostasis than beef tallow under overnutrition conditions. (A–E) Glucose homeostatic parameters in mice fed beef tallow or lard as 45 *kcal% fat* (n = 5/group). Fasting and fed blood glucose levels (A), glucose tolerance test (B), insulin tolerance test (C), plasma insulin levels (D), and plasma GLP-1 levels (E). *p < 0.05, **p < 0.01 for one-way ANOVA followed by Tukey’s multiple comparisons in (A); two-way ANOVA followed by Sidak’s multiple comparison tests in (B) and (C), t-tests in (D) and (E). Data represent the mean ± SEM.

Excess nutrient induced by a HFD is associated with glucose intolerance and insulin resistance. Thus, we further investigated whether the whole-body glucose metabolism was affected in lard-fed mice by measuring blood glucose levels and performing a glucose tolerance test and an insulin tolerance test compared with beef tallow-fed mice. As shown in Fig 2A, the lard-fed group exhibited significantly lower fasting and fed blood glucose levels than the beef tallow-fed group. Furthermore, the lard-fed group exhibited significantly lower glucose levels after glucose and insulin administration than the beef tallow-fed group, suggesting that lard-fed mice had better glucose tolerance and insulin sensitivity (Fig 2B and C). Consistent with higher glucose tolerance and insulin sensitivity, lard-fed mice displayed significantly lower blood insulin levels and higher blood GLP-1 levels than beef tallow-fed mice under overnutrition (Fig 2D and E).

### Lard-fed mice exhibited lower food intake than beef tallow-fed mice under overnutrition conditions

**Fig 3:**
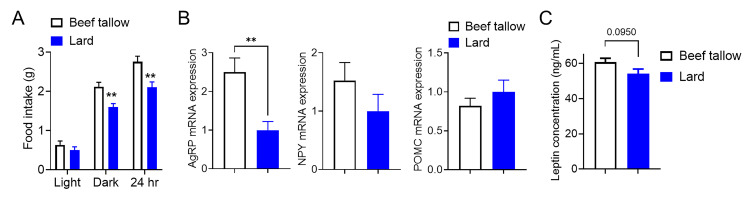
Lard intake reduces food intake compared with beef tallow intake. (A) Food intake of beef tallow-fed and lard-fed mice during 12-h light and dark phases (n = 6/group). (B) Hypothalamic mRNA expression of the feeding-related neuropeptide genes. Hypothalami were collected from lard and beef tallow-fed male mice (n = 5/group). qPCR analyses were performed to measure mRNA. (C) Plasma leptin levels of beef tallow-fed and lard-fed mice. **p < 0.01 for t-tests in (A) and (B). Data represent the mean ± SEM.

Next, to measure food intake, the mice were housed in a food intake measurement system. The lard-fed mice consumed significantly lower amounts of food during the active (dark) phase and total 24 h (Fig 3A), which was associated with decreased expression of orexigenic neuropeptide *AgRP* mRNAs compared with beef tallow-fed mice (Fig 3B). Additionally, lard-fed mice showed slightly lower plasma leptin levels than beef tallow-fed mice under body weight-matched conditions (Fig 3C). Thus, decreased food intake and preferential oxidation of fat as an energy substrate likely contributed to the decreased body weight in lard-fed mice compared with beef tallow-fed mice under hypercaloric feeding.

### Lard-fed mice exhibited higher hypothalamic leptin responsiveness than beef tallow-fed mice under overnutrition conditions

**Fig 4:**
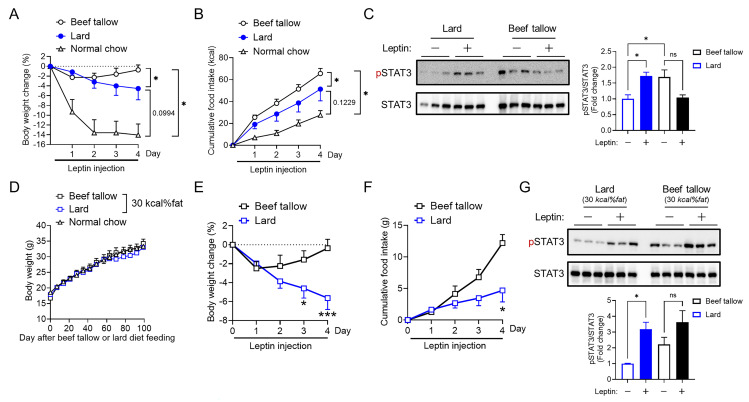
Leptin sensitivity is higher in lard intake than in beef tallow intake under overnutrition conditions. (A and B) Male mice were maintained on a lard or beef tallow diet of 45 *kcal% fat* for four months and administrated with leptin (0.5 μg/mouse, ICV for four days) once a day at 5 p.m. during the indicated period. Age- and body-weight-matched cohorts were used (n = 5/group). Body weight change (A) and cumulative food intake (B). (C) Western blot images (left) and quantification (right) of hypothalamic STAT3 (Tyr^705^) phosphorylation 1 h after a bolus injection of leptin (0.5 μg/mouse, ICV) or saline into beef tallow or lard-fed mice (n = 3/group). (D) The daily body weight of mice fed with beef tallow or lard as 30 *kcal% fat*, and normal chow as 10 *kcal% fat* over an indicated period (n = 4–6/group). (E and F) Male mice were maintained on a lard or beef tallow diet at 30 *kcal%* *fat* for four months and administrated with leptin (0.5 μg/mouse, ICV for four days) once a day at 5 p.m. during the indicated period. (G) Western blot images (left) and quantification (right) of hypothalamic STAT3 (Tyr^705^) phosphorylation 1 h after a bolus injection of leptin (0.5 μg/mouse, ICV) or saline into mice fed beef tallow or lard diet as 30 *kcal% fat* (n = 3/group). *p < 0.05, ***p < 0.001 for two-way ANOVA followed by Sidak’s multiple comparisons tests in (A), (B), (E) and (F), one-way ANOVA followed by Tukey’s multiple comparisons test in (C) and (G). Data represent the mean ± SEM.

In this study, we confirmed that lard-fed mice showed significantly lower body weight gain (Fig 1A) and food intake (Fig 3A) and slightly lower plasma leptin levels (Fig 3C) compared with beef tallow-fed mice for body weight-matched cohorts. These results suggested that lard-fed mice exhibited better leptin responsiveness. To assess this hypothesis, we performed ICV injections of leptin for four days and evaluated the body weight and food intake changes in body weight-matched lard-fed or beef tallow-fed cohorts (after HFD feeding, beef tallow: 40.8 ± 0.9 g versus lard: 40.5 ± 1.0 g, p > 0.05 based on t-tests). Central leptin administration significantly reduced the body weight of lard-fed mice, whereas leptin failed to reduce the body weight of beef tallow-fed mice (Fig 4A). Cumulative food intake under leptin treatment was decreased in lard-fed mice compared to beef tallow-fed mice (Fig 4B). Since basal food intake of lard-fed mice was significantly decreased compared to beef tallow-fed mice (Fig 3A), we investigated leptin responsiveness further by quantifying leptin-induced STAT3 phosphorylation using western blotting. The phosphorylation of STAT3 is a part of leptin signaling and is well-established as a marker of activated leptin signaling. ICV leptin or vehicle injections were administered 1 h before the mice were sacrificed. Leptin-induced phosphorylation of STAT3 was significantly increased in lard-fed mice (Figs 4C and S1) but not in beef tallow-fed mice (Figs 4C and S1). Collectively, instead of beef tallow-fed mice showing decreased leptin responsiveness, lard-fed mice responded to leptin with body weight reduction, suppression of food intake, and increased phosphorylation of STAT3 in the hypothalamus. This indicates that lard intake might be able to maintain hypothalamic leptin signaling better than beef tallow intake under overnutritional conditions.

To further support the improved leptin sensitivity in lard-fed mice under obesogenic conditions, we fed them a 30 *kcal% fat* diet, which is a more physiological fat intake condition. The body weight of lard-fed as 30 *kcal% fat* mice did not differ from that of beef tallow-fed as 30 *kcal% fat* mice after 100 days of feeding (Fig 4D), and both fat conditions were equivalent to the *10 kcal% fat* condition in terms of body weight gain. However, when leptin was infused into the brains of lard-fed or beef tallow-fed mice under conditions of 30 *kcal% fat* with no difference in body weight between the groups, lard-fed mice were markedly sensitized to leptin-responsive neurons, as indicated by the restoration of leptin-induced suppression of food intake, reduction in body weight (Fig 4E and F), and phosphorylation of the leptin signaling mediator STAT3 (Figs 4G and S2). These findings suggest that lard-fed mice exhibit higher leptin sensitivity than beef tallow-fed mice under normal body weight conditions.

### The effect of lard intake on energy balance is abolished in *ob/ob* mice

**Fig 5:**
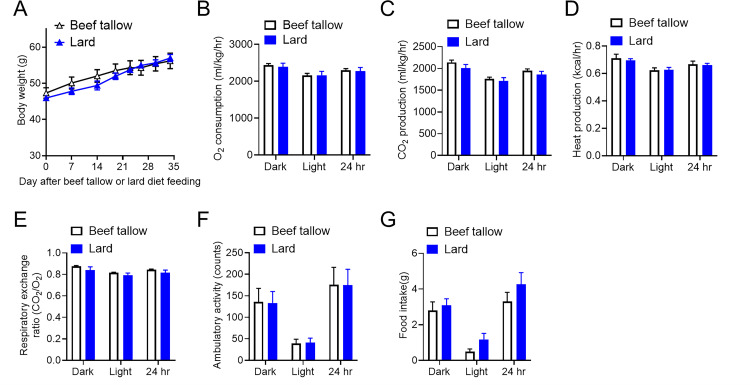
Metabolic phenotypes of beef tallow-fed and lard-fed *ob/ob* mice. (A) The daily body weight of *ob/ob* male mice fed with beef tallow or lard as 45 *kcal% fat* over an indicated period (n = 4/group). Male *ob/ob* mice were maintained on a lard or beef tallow diet as 45 *kcal% fat*. (B–G) Metabolic profile of beef tallow-fed and lard-fed *ob/ob* mice (n = 4/group) for O_2_ consumption (B), CO_2_ production (C), heat production (D), respiratory exchange ratio (E), ambulatory activity (F), and food intake (G) during 12-h light and dark phases after HFD feeding. Age- and body-weight-matched cohorts were considered (Lard-fed *ob/ob*: 57.00 ± 0.98 g vs. beef tallow-fed *ob/ob*: 56.21 ± 2.13 g, p > 0.05, t-tests) in the CLAMS study. Data represent the mean ± SEM.

Because lard-fed mice showed reduced body weight gain and respiratory quotient compared with beef tallow-fed mice, the next step was to determine whether the increase in energy balance due to lard consumption was mediated by leptin signaling. We fed beef tallow and lard as 45 *kcal% fat* to *ob/ob* mice, which is another mouse model of obesity. There were no differences in body weight between the two groups (Fig 5A). Furthermore, there were no significant differences in O_2_ consumption, CO_2_ production, heat production, respiratory exchange ratio, activity, or food intake between *ob/ob* mice fed lard and beef tallow (Fig 5B–G). These results suggest that leptin signaling may contribute to the lard-induced reduction in body weight gain, suppression of food intake, and preferential oxidation of fat as an energy substrate under hypercaloric feeding.

### Lard-fed mice exhibited lower expression of hypothalamic *SOCS3* than beef tallow-fed mice

**Fig 6:**
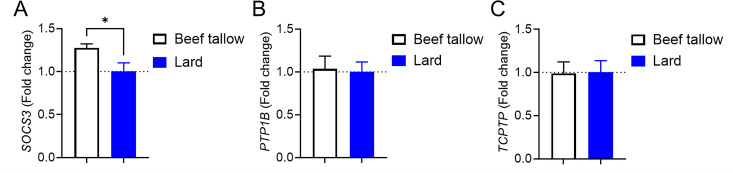
Hypothalamic expression of genes involved in leptin responsiveness in beef tallow-fed and lard-fed mice under overnutrition conditions. Hypothalami were collected from male mice fed lard or beef tallow for five months. *SOCS3* (A), *PTP1B* (B), and *TCPTP* (C) mRNA expression. Age- and body weight-matched cohorts were used (n = 4/group). *p < 0.05 for t-tests in (A). Data represent the mean ± SEM.

To elucidate the mechanism underlying increased hypothalamic leptin responsiveness in lard-fed mice, we assessed the expression levels of factors related to decreased leptin responsiveness. SOCS3 [11–13], PTP1B [25–27], and TCPTP [28] have been identified as crucial mediators of leptin resistance. All these factors are upregulated in the hypothalamus by HFD-induced obesity, and neuronal deletion of these inhibitors protects against HFD-induced obesity, leptin resistance, and insulin resistance. Thus, SOCS3 and tyrosine phosphatases play multiple roles in HFD-induced obesity. Therefore, we chose these mediators as major negative regulators of leptin signaling in the hypothalamus. *SOCS3* was expressed at significantly lower levels in lard-fed mice than in beef tallow-fed mice (Fig 6A). The other factors were not significantly different (Fig 6B and C). These findings suggest that lard intake is better at protecting against dietary obesity and metabolic disruption than beef tallow intake under overnutritional conditions, by controlling food intake and maintaining hypothalamic leptin responsiveness.

## Discussion

In this study, we demonstrated that lard intake is better than beef tallow intake in maintaining hypothalamic leptin responsiveness and glucose balance under overnutritional conditions. Overnutrition is associated with reduced sensitivity to metabolic hormones such as leptin in the hypothalamus. It is important to recognize leptin as an adiposity signal and a critical hormone of energy balance, and circulating leptin levels are directly correlated with body mass index [1,29,30]. Furthermore, local administration of leptin into the brain reduces food intake and body weight [31–33]. Although the hypothalamus is an important regulator of systemic metabolism, excess nutrients can reduce the hypothalamic response to leptin, thereby promoting diet-induced obesity. Therefore, the hypothalamus is involved in the biological response to leptin resistance under obesogenic conditions.

Obesity is a global health concern in children and adults and is associated with serious comorbidities, including type 2 diabetes, hypertension, cardiovascular disease, certain cancers, and reduced life expectancy. Reduction in body weight has beneficial effects on several metabolic risk factors. The first report demonstrating that HFD induced obesity was published in 1959 [34]. Subsequent studies have revealed that HFDs promote hyperglycemia, hyperleptinemia, whole-body insulin resistance, and hypothalamic leptin resistance [1,2,35–39]. Therefore, it is necessary to identify nutritional conditions or orally active compounds that increase leptin sensitivity in the central nervous system.

Beef tallow and lard are recognized as major edible animal fats. However, there is a lack of reports that directly compare the effects of commonly used animal fat sources on the development of diet-induced obesity and leptin resistance. To clarify the physiological importance of the source and composition of fat in commonly used experimental HFDs, we investigated the effects of lard and beef tallow diets on energy balance and hypothalamic leptin responsiveness. In the present study, we demonstrated that lard-fed mice showed lower body weight gain than beef tallow-fed mice under overnutrition conditions. Additionally, lard-fed mice exhibited better glucose tolerance, systemic insulin sensitivity, and higher levels of GLP-1 secretion under overnutrition. This is consistent with previous reports showing that GLP-1 levels and secretion increased during obesity development protected against glucose intolerance induction, and GLP-1 treatment improved insulin sensitivity in rodents fed an HFD [40–42]. Collectively, these results suggest that high GLP-1 levels in lard-fed mice are involved in maintaining glucose balance and insulin sensitivity under overnutrition.

We also revealed that mice fed lard were more protected against diet-induced hypothalamic leptin resistance than mice fed beef tallow under overnutritional conditions. The decrease in body weight gain in lard-fed mice compared to beef tallow-fed mice was likely due to hypophagia, without changes in energy expenditure, because motor activity and thermogenesis were unchanged. Consistent with reduced feeding behavior, lard-fed mice also showed reduced mRNA levels of the orexigenic neuropeptide *AgRP* in the hypothalamus. Additionally, lard-fed mice had a decreased respiratory quotient, suggesting the preferential use of fat as an energy source over beef tallow. This may also contribute to reduced body weight gain.

In addition to its effect on energy balance, lard intake resulted in better hypothalamic leptin responsiveness than beef tallow intake during overnutrition. Lard-fed mice showed lower plasma leptin levels, fat mass and food intake than beef tallow-fed mice, indicating enhanced leptin sensitivity under obesogenic conditions. Compared with mice fed a normal chow as 10 *kcal% fat*, lard-fed as 45 *kcal% fat* mice showed a decreasing trend in leptin sensitivity, whereas beef tallow-fed mice exhibited a significant decrease in leptin sensitivity. We further demonstrated that the central administration of leptin significantly suppressed food intake, reduced body weight, and enhanced phosphorylation of the leptin signaling mediator STAT3 only in mice fed lard compared with mice fed beef tallow under overnutrition conditions. The baseline pSTAT3 levels in the hypothalamus (without leptin injection) were significantly lower in lard-fed mice than in beef tallow-fed mice under obesogenic conditions (Fig 4C). Differences in baseline pSTAT3 levels may represent plasma leptin levels and systemic leptin sensitivity, suggesting that lower pSTAT3 levels in lard-fed mice indicate higher leptin responsiveness. To further confirm the improved leptin responsiveness, leptin sensitivity was measured under 30 *kcal% fat* conditions. Remarkably, even under 30 *kcal% fat* conditions, which did not induce any difference in body weight, we found that lard-fed mice exhibited higher leptin sensitivity than tallow-fed mice. In this study, we have performed central administration of leptin into lard or beef tallow-fed mice. Since the leptin resistance involved not only a lack of responsiveness of target neurons in the hypothalamus but also altered transport of leptin from the periphery to the brain, it is need to clarify the effect of the blood-brain barrier in these diet conditions. Further studies will thus need to experimentally confirm whether lard or beef tallow-fed mice directly affects transport of leptin from the periphery *in vivo*. Collectively, these results support our hypothesis that lard intake is more likely to maintain hypothalamic leptin sensitivity than beef tallow intake.

In this study, we did not observe a difference in body weight between lard-fed *ob/ob* mice and beef tallow-fed *ob/ob* mice, possibly because of the late onset of the HFD challenge or because body weight may have reached a plateau at a young age. We speculated that there is a relationship between lard intake and leptin signaling since the enhancement of energy metabolism by lard intake in adult mice was abolished in *ob/ob* mice. However, further studies are required, as it is possible that another phenotype may be obtained by feeding lard or beef tallow to *ob/ob* mice during the young growth period.

SOCS3 has been identified as a crucial mediator that induces leptin resistance by directly inhibiting leptin signaling [11–13]. SOCS3 is also known to be upregulated in the hypothalamus by diet-induced obesity [11,26–28]. Moreover, the neuron-specific deletion of SOCS3 protected against diet-induced obesity, leptin resistance, and insulin resistance [12,13,25,28]. Thus, SOCS3 may contribute to the development of diet-induced obesity and hypothalamic leptin resistance. We showed that a lard-based diet resulted in the suppression of a direct endogenous inhibitor of cellular leptin signaling, SOCS3, and reductions associated with lard intake likely account, at least in part, for enhanced leptin sensitivity.

One of the most important issues arising from this study was the difference in leptin sensitivity between beef tallow and lard intake. In general, animal products have a high-fat content, which is associated with an increased consumption of long-chain saturated fatty acids, especially palmitic and stearic acids. These saturated fatty acids alter the hypothalamic control of energy homeostasis by inducing hypothalamic inflammation and insulin and leptin resistance, thereby promoting obesity [43]. Both lard and tallow contain approximately 24% palmitic acid, but palmitic acid is distributed differently in their triglyceride component molecules [20]. Whereas more than 70.5 mol% of palmitic acid in lard is located in the sn-2 position, less than 15 mol% of palmitic acid in beef tallow is located in the sn-2 position [21–23]. Pancreatic lipase has specificity for the TG sn-1,3 ester bond and hydrolyzes TG to generate sn-2 monoacylglycerols and free fatty acids. Therefore, beef tallow may produce more free palmitic acid than does lard. Previous studies have shown that elevated palmitic acid concentrations in the brain promote obesity and obesity-related metabolic disorders by impairing hypothalamic leptin signaling [19,44,45]. Additionally, we have recently shown that 2-monopalmitin (β-palmitate), but not 1-monopalmitin, enhances hypothalamic leptin responsiveness in *ex vivo* brain slices and diet-induced obese mice [24]. Most importantly, we found that peripheral injection of 2-monopalmitin enhanced the anorectic effect of centrally administered leptin [24]. Within the hypothalamic nuclei, the arcuate nucleus of the hypothalamus, which densely expresses the leptin receptor and is adjacent to the third ventricle, has been described as having a weak and incomplete blood-brain barrier, which potentially allows access to peripherally derived circulating bioactive molecules, nutrients, and gut hormones via the bloodstream [46,47]. Although the mechanism by which 2-monopalmitin is delivered to the brain remains unclear, we showed that central administration of 2-monopalmitin increases energy expenditure, decreases the respiratory quotient, and improves leptin and insulin sensitivity. Thus, we speculate that exogenous leptin administered to the brain in lard-fed mice causes a more robust and immediate reduction in body weight and food intake than in beef tallow-fed mice due to the 2-monopalmitin-induced enhancement of hypothalamic leptin responsiveness and the reduced induction of inflammatory responses by the release of palmitic acid under lard-fed conditions. While we reported the beneficial effects of lard intake on energy balance compared with beef tallow intake under obesogenic conditions, the 2-monopalmitin project is in its preliminary stages, and we believe this interesting aspect is worth further investigation. Moreover, a recent study has shown that oral administration of food-derived components enhanced leptin-induced STAT3 phosphorylation in the hypothalamus *in vitro* and *in vivo* [19]*.* Therefore, the possibility that lard contains a novel functional component that enhances leptin sensitivity in the hypothalamus cannot be discounted. Further studies are necessary to clarify the components of lard that affect leptin sensitivity in the hypothalamus.

In summary, our results suggest that lard intake is better than beef tallow intake for maintaining hypothalamic leptin responsiveness and glucose balance under overnutritional conditions, such as obesogenic or diabetic conditions.

## Supporting information

S1 Fig Raw image for Fig 4C. (PPTX)

S2 Fig Raw image for Fig 4G. (PPTX)
