# Supplementary figures and images for: Lard intake results in better hypothalamic leptin responsiveness than beef tallow intake during overnutrition
Source: PLoS One. 2025 Jul 21;20(7):e0326847. doi: 10.1371/journal.pone.0326847 (PMC12279138; doi:10.1371/journal.pone.0326847)

## Slide 1
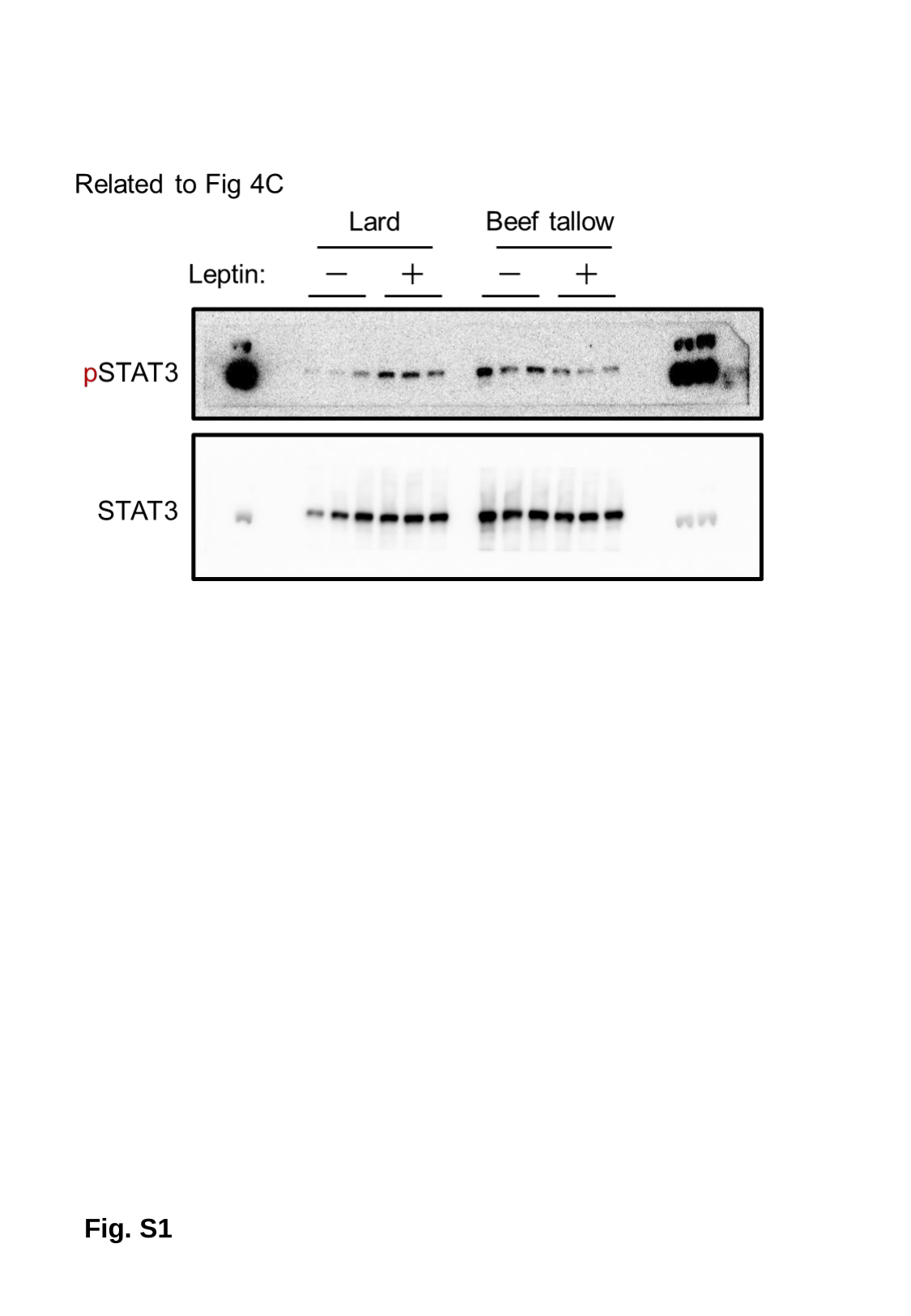

Fig. S1

Supplement: S1 Fig — (PPTX) [file pone.0326847.s001.pptx]

## Slide 1
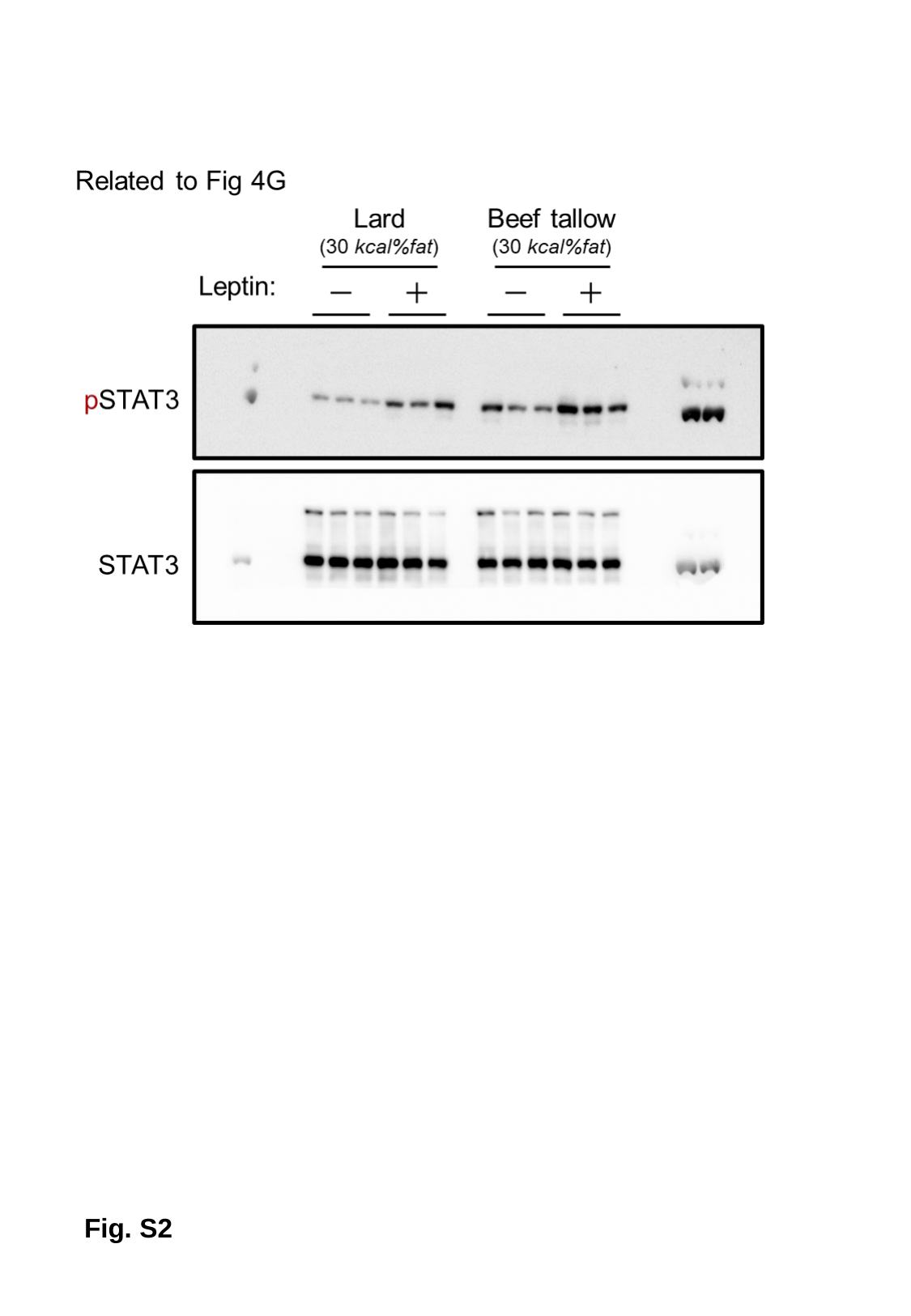

Fig. S2

Supplement: S2 Fig — (PPTX) [file pone.0326847.s002.pptx]
